# Circadian Variation in Vasoconstriction and Vasodilation Mediators and Baroreflex Sensitivity in Hypertensive Rats

**DOI:** 10.5334/jcr.185

**Published:** 2019-10-14

**Authors:** Tamar Kezeli, Nikoloz Gongadze, Galina Sukoyan, Marina Shikhashvili, Zaza Chapichadze, Maia Okujava, Nino Dolidze

**Affiliations:** 1 Department of Pharmacology, Faculty of Medicine, I. Javakhishvili Tbilisi State University, Tbilisi, GE; 2 Department of Medical Pharmacology, Tbilisi State Medical University, Tbilisi, GE; 3 Department of Molecular and Clinical Pharmacology, International Scientific Centre of Introduction of New Biomedical Technology, Tbilisi, GE; 4 David Tvildiani Medical University, Tbilisi, GE; 5 State Regulation Agency for Medical Activities, Tbilisi, GE

**Keywords:** circadian fluctuation, hypertension, calcitonin gene-related peptide, endothelin-1, epoxyeicosatrienoic acids, Baroreflex sensitivity, hemodynamic

## Abstract

The purpose of this study was to evaluate the relationship between the circadian profile of the vasorelaxing substances calcitonin gene-related peptide (CGRP) and epoxyeicosatrienoic acids (EETs) and the vasconstrictive agent endothelin-1 (ET1) and the daily rhythms of cardiac hemodynamic indices (CHI) and baroreflex (BRS) in Wistar rats with 1 kidney-1 clip model of arterial hypertension (1K-1C AH). The animals were divided into 3 groups: I- sham-operated (SO), II- 4-week and III- 8-week 1K-1C AH rats. Plasma concentration of ET1, CGRP and EET’s were investigated every 4 h. In conscious freely moving 1K-1C AH rats unlike SO animals blood pressure (BP), heart period (HP) and BRS underwent significant circadian fluctuations, with more marked increase in mean values of BP in 8-week hypertensive rats in comparison to 4-week hypertensive rats (179 ± 5 vs. 162 ± 4 mm Hg, p < 0.05). These alterations correlated with more significant reduction in HP (138 ± 5 vs. 150 ± 6 ms, p < 0,05) and BRS (0.44 ± 0.04 vs. 0.58 ± 0.04 ms mm Hg^–1^, p < 0.05) in 8-week 1K-1C AH rats. The acrophases of BP in 8-week 1K-1C AH rats in comparison with 4-week were shifted to more late night hours (1:58 a.m. vs. 11:32 p.m.) and in both groups of animals corresponded to lowest circadian plasma levels of CGRP and EETs and to greatest level of ET1. SO rats were characterized by lower values of BP (121 ± 3 mm Hg, p < 0,05) and higher indices of HP (158 ± 2 ms, p < 0,05) and BRS (0.86 ± 0.02 ms mmHg^–1^, p < 0,001) in comparison with 1K-1C AH rats 4-week duration. The acrophases of BP, HP and BRS in hypertensive animals were revealed at 14.8 ± 0.5 h, 13.6 ± 0.4 h and 13.1 ± 0.2 h, which correlated with maximal circadian contents of ET1 and CGRP at 24:00 h and EETs at 12:00 h and were shifted in comparison to sham-operated group. In rats with 1K-1C AH, plasma levels of ET1, CGRP and EETs undergo circadian fluctuation with corresponding alterations in CHI and BRS which are more markedly expressed on the late stage of diseases and could be used in future for predictive, preventive, and personalized treatment of arterial hypertension.

## Introduction

The association between diurnal variation of the heart rate, blood pressure (BP), vascular tone, and QT interval and predisposition to cardiovascular disease has been noted in both normotensive and hypertensive individuals. Despite a broad range of evidence that the endogenous circadian clock is involved in the regulation of blood pressure, cardiac hemodynamic and baroreflex sensitivity (BRS), the full molecular mechanisms of such regulation, disruption and efficacy of therapies for its correction remain unclear [1,2,3,4,5]. The circadian variation in vascular events corresponds to that in blood pressure and to the oscillation of genes relevant to hemostasis, but whether this reflects an important role for the molecular clock or merely the physical and emotional stress remains unknown. The presence of a close correlation between the circadian rhythm of BP and glomerular filtration rate diseases and decrease in creatinine clearance in patients with chronic kidney led to the conclusion that lack of nocturnal dipping (i.e. typically considered a nocturnal decline of 10% or more [3]) could be independent predictive factors of cardiovascular events and target organ damage [6,7]. Moreover, it was assessed that the nighttime BP and the dipping are associated with the circadian pattern of sodium excretion and strongly coupled with disturbances of the ET system [2,7,8,9,10,11]. The ET system consists of two G protein coupled-receptors, ETA and ETB, and three endogenous ligands, ET-1, ET-2, and ET-3 [12,13]. Experimental and clinical data have provided evidence that hyperproduction of the vasoconstrictive component ET1, which reduces coronary blood flow even in the absence of detectable vasospasm of large epicardial arteries, plays an important role in the pathogenesis of atherosclerosis, for which hypertension is an important risk factor, and in ischemic heart disease and stroke, pulmonary hypertension, and vasculitis. The pressor effects of hyperproduction of ET1 could trigger counterregulatory mechanisms responsible for maintenance of adequate BP regulation, as ET1 plasma concentration was found to be higher in hypertensive patients as compared to normotensive controls [6,8,10,11]. A few studies suggest that ET1 could be a key mediator in the 24-hour control of blood pressure but its role in circadian variability of hemodynamic parameters during progression of vasorenal hypertension has not been elucidated [10,11,12]. The functional adaptive mechanism of regulation of vascular tone includes enhancing of endothelium-derived NO release, which can reduce ET1-induced vasoconstrictions and inhibit the production of ET1, and the vasodilatory substances calcitonin gene-related peptide (CGRP) and epoxyeicosatrienoic acids (EETs), which reduce ET1 induced vasoconstriction and prevent ET1-induced increases in BP [14,15,16,17]. Dose-related reduction in blood pressure in hypertensive animals, caused by administration of CGRP, suggests its possible implication in blood pressure regulation [18]. Studies have shown that time of day variation influences endothelium-dependent vasodilation as a cardioprotective mechanism against potentially adverse time dependent changes in cardiac hemodynamics indices (CHI). However, the relationship between circadian profile of CGRP in different forms of vasorenal hypertension is not fully understood [19,20,21,22]. EETs are arachidonic acid metabolites that contribute to vascular and cardiac physiology [23,24,25,26,27]. It was determined that administration of EETs resulted in increase in organ blood flow or vascular diameters, lowering blood pressure in a number of experimental models of hypertension. Vascular action of EETs is influenced by soluble epoxide hydrolase (sEH) that degrades EETs to diols [28]. Early studies provide evidence that EETs exert vasodilatory action by the activation of vascular smooth muscle cells large-conductance calcium-activated K^+^(KCa) channels [27,28,29,30]. The rhythmic production of multiple types of EETs was observed in endothelial cells and astrocytes [31] and possibly contribute to circadian changes in blood flow and alter risk of adverse cardiovascular events throughout the day [32,32,33,34,35]. However data concerning the circadian profile of these substances and involvement in the diurnal variability of CHI and BRS in primary or secondary arterial hypertension is limited and difficult to extrapolate into clinical practice. The purpose of this study was to evaluate the relationship between the circadian profile of plasma levels of ET1, CGRP and EETs and circadian rhythms of CHI and BRS in the 1 kidney-1 clip (1K-1C) model of hypertension in rats, which is characterized by modest changes in renin and pronounced reduction in glomerular filtration rate, sodium retention and high volume [36–37].

## Materials and Methods

### Animals and experimental study design

We used 105 male Wistar rats, 12-week-old, weighing 250–300 g. The animals were handled in compliance with the European Convention on Animal care and received human care (Official Daily N.L358/1-358/6, 18 December 1986) and compliance with ethical standards and approved by the Animal Care Committee of the Department of Pharmacology of I. Javakhishvili Tbilisi State University and the Department of Medical Pharmacology of Tbilisi State Medical University. Animals were maintained and fed ad libitum on the standard normal-protein diet and had free access to water. They were housed individually in humidity and temperature-controlled room with a 12-h light/dark cycle at a temperature of 22°C. All animals were randomized into 3 groups: I control – sham operated (SO); II and III – main groups containing rats with 1K-1C atrial hypertension (1K-1C AH) 4-week and 8-week, respectively. To induce 1K-1C hypertension (n = 70), a right nephrectomy was performed with a constricting silver clip with an internal diameter 0.2 mm on the renal artery of remaining kidney under pentobarbital (40 mg/kg, i.p.) anesthesia [38]. Control (SO, n = 35) rats surgical procedures were carried out only with kidney exposure without its removal or renal artery ligation. After a 3-week observation period, mean arterial blood pressure was measured and blood samples were collected.

### Circadian rhythm, cardiac hemodynamics and baroreflex sensitivity studies

The tail cuff method was used to confirm the development of arterial hypertension. Animals were involved in the study 4 and 8 weeks after the surgical procedure. Three days before experiments in anesthetized hypertensive rats polyethylene catheters were placed in the right jugular vein for drug administration and in the right femoral artery connected to a blood pressure transducer for measuring blood pressure (BP) with an electromanometer and heart period (HP) with a cardiotachometer. Circadian rhythm was studied during three days with a preliminary 24-h monitoring of corresponding CHI, BRS and acrophases obtained by cosinor analyses [39]. The assessment of rhythmic characteristics of the studied parameters include [40] the mesor (middle value of the fluctuated corresponding indices), the amplitude (half the difference between the maximal and minimal value) and the acrophase (time in hours and minutes expressing peak value of fitted parameter). Baroreflex sensitivity (BRS) was assessed by measuring HP in response to rises in BP 20–30 mm Hg above control after the i.v. injection of phenylephrine (3–10 mcg/kg), and the relationship slope of BP and HP as an index of BRS was determined as described below [40–41].

### Investigation of biochemical markers

Plasma content of ET1, CGRP and EETs were investigated every 4 h (n = 7, each time point) in plasma blood taken from the femoral artery (placed in tubes with 1% heparin) and the animals were euthanized. Levels of CGRP were determined using the commercial available ELISA kit (Cayman Chemical, Michigan, USA) and EETs levels were analyzed by an ELISA kit (Detroit R&D Inc., Detroit, MI, USA) in according with the manufacturer’s instruction. ET1 was measured by R&D Systems for Human endothelin-1 Immunoassay (Great Britain) as described earlier [42].

### Statistical analyses

Results are reported as mean ± SD (standard deviation) and data were analyzed statistically. All data were compared by Student’s *t* test or analysis using repeated-measures ANOVA for multiple comparisons using SPSS (SPSS Inc., IBM). A two-sided *P* value of 0.05 was considered statistically significant.

## Results

### Circadian variation of cardiac hemodynamics indices and baroreflex sensitivity in hypertensive rats

**Figure 1:**
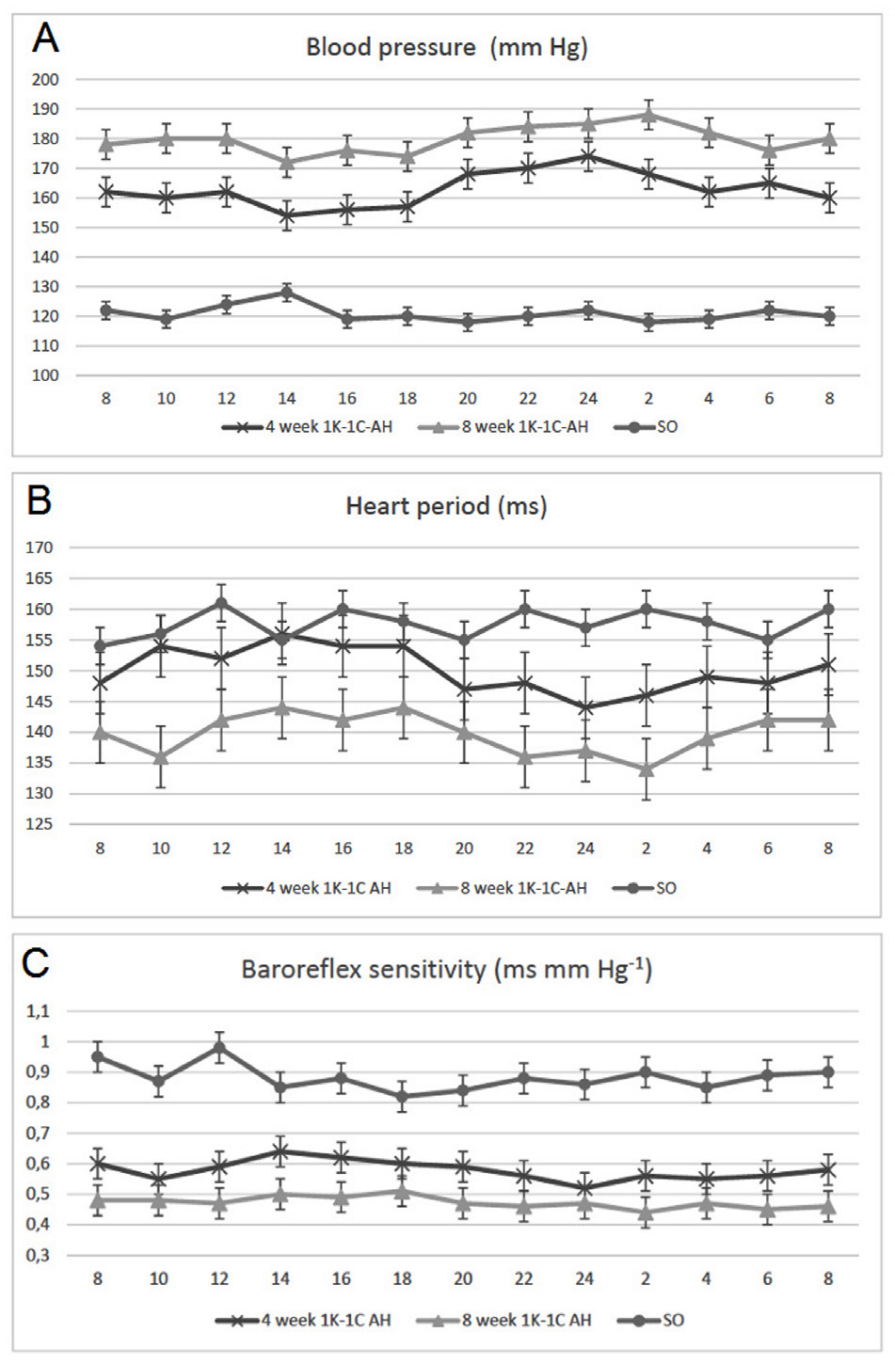
Circadian variations of blood pressure **(A)**, heart period **(B)** and baroreflex sensitivity **(C)** in 1K-1C arterial hypertension and sham-operated (SO) rats.

The analysis of CHI in conscious freely moving animals showed that there were significant differences in baseline values of BP, HP and BRS in 4 and 8-week hypertensive rats. In 4-week hypertensive rats the mean value of BP was 162 ± 8 mmHg, which correlated with mean significances of HP – 150 ± 4 ms and BRS – 0.58 ± 0.04 ms mm Hg^–1^. In 8-week hypertensive rats in comparison to 4-week hypertensive rats the mean value of BP was markedly increased (179 ± 5 mm Hg. p < 0.05), associated with decreased HP (138 ± 6 ms, p < 0.05) and blunted BRS (0.44 ± 0.02 ms mm Hg^–1^, p < 0.05). In both groups of animals, CHI and BRS underwent diurnal variation with circadian acrophases of BP in 4 and 8-week hypertensive rats (174 ± 4 mm Hg vs. 188 ± 8 mm Hg, p < 0.05) in nighttime at 23:32 h and 01:58 h, while its lowest level was revealed in the light period (156 ± 6 mm Hg vs. 170 ± 8 mm Hg) between 14:00 h and 18:00 h, respectively (Figure 1). Such changes in diurnal rhythm of BP in hypertensive rats were correlated with alterations of circadian values of HP and especially BRS, which acrophases in 4 and 8-week hypertensive rats (0.64 ± 0.02 ms mm Hg^–1^ vs. 0.51 ± 0.04 ms mm Hg^–1^) in contrast to BP occurred in daytime at 13:58 h and 17:40 h, respectively. Eventually, according to our results, it may be suggested that diurnal rhythm of CHI and BRS in hypertensive rats is characterized by circadian profile, associated with circadian acrophases of BP at late nighttime, and circadian disorders of relationship between acrophases for BP, HP and BRS more significantly expressed in 8 week hypertensive rats.

### Circadian profile in ET1 plasma concentracion in rats with hypertension

The analysis of ET1 plasma concentration in blood samples obtained at 8:00, 12:00, 16:00, 20:00 and 24:00 h revealed a circadian profile in each of group of animals (Table 1). Circadian clock fluctuation of ET1 in blood plasma of SO rats was characterized by peak values at 16–20 h. The mean daily plasma content of ET1 in 4-week 1K-1C AH exceeded the level in SO group 1.6 fold, and in 8-week 1K-1C-AH 2.4 fold. Moreover, the acrophases for ET1 in plasma in 4-week and 8-week 1K-1C-AH were shifted to a late night time – 24:00 h, indicating an increased formation of vasoconstrictor component in nocturnal time, especially in 8-week 1K-1C AH.

**Table 1:** Circadian profile of endothelin-1, epoxyeicosatrienoic acids (EETs) and calcitonin generelated peptide (CGRP) in plasma of rats with 1K-1C model of arterial hypertension (1K-1C AH).

Hours	Endothelin-1, fmol/ml			EETs, ng/ml			CGRP, pg/ml		

	SO	1K-1C AH, week		SO	1K-1C AH, week		SO	1K-1C AH, week	

		4	8		4	8		4	8

8:00	1.0 ± 0.2	2.0 ± 0.2***	3.5 ± 0.2***###	15.2 ± 0.6	12.1 ± 1.6**	10.0 ± 0.8***##	68.6 ± 3.5	61.4 ± 3.4*	52.4 ± 2.9***###
12:00	1.4 ± 0.2	2.2 ± 0.2**	3.0 ± 0.4***##	15.6 ± 1.0	13.5 ± 1.2*	10.6 ± 0.7***#	69.0 ± 4.6	58.0 ± 3.6**	48.8 ± 2.5**###
16:00	1.9 ± 0.2	1.6 ± 0.2	2.8 ± 0.4**###	17.4 ± 1.5	14.9 ± 0.9*	11.6 ± 0.5**###	72.8 ± 5.4	54.2 ± 3.2***	45.2 ± 3.4***#
20:00	l.8 ± 0.2	3.2 ± 0.3***	4.2 ± 0.25***##	18.0 ± 1.2	12.5 ± 0.8***	9.5 ± 0.4***##	76.0 ± 6.5	52.5 ± 3.8***	44.0 ± 3.2***#
24:00	1.6 ± 0.3	3.4 ± 0.3***	4.8 ± 0.4***##	18.6 ± 1.8	11.8 ± 1.0***	8.2 ± 0.6***###	80.4 ± 8.2	50.2 ± 2.8***	41.8 ± 2.0***##
Mean	1.54 ± 0.22	2.5 ± 0.2***	3.7 ± 0.3***###	17.0 ± 1.2	13.0 ± 1.0***	10.6 ± 0.6***#	73.4 ± 5.6	55.3 ± 3.0	46.4 ± 2.6##

*Note*: Significance of difference of comparison: * – with SO group, # – with 1K-1C AH 4-weeks group; one symbol – p < 0.05, two – p < 0.01, three – p < 0.001.

The analyses of our results showed that the high production of ET1 at late night hours coincided with acrophases of BP in both group of animals, while ET1 relatively low circadian plasma level was correlated with a light period associated with smallest values of BP. That could suggest the correlation between circadian production of ET1 and diurnal variability of CHI in hypertensive rats.

### Circadian profile of EETs plasma concentration in rats with hypertension

EETs mean plasma content in 4-week 1K-1C AH decreased about 1.3 fold and in 8-week 1K-1C AH about 1.6 fold in relation to control values (SO group). The analysis of circadian fluctuation of EETs in plasma revealed the acrophases at a similar time of day – at 16:00 h with a reduction in their diurnal production at 24:00 h in 1K-1C AH.

### Circadian profile of CGRP plasma concentration in rats with hypertension

The mean plasma level of CGRP in SO animals exceeded the level of the peptide in 4-week 1K-1C AH 1.3 fold and in 8-week 1K-1C-AH 1.6 fold. The diurnal variability in plasma CGRP with corresponding acrophases in 4-week and 8-week 1K-1C AH occurred in the light time at 16:00 h, while in SO at a late night time – 24:00 h. According to our results, it may be postulated that the circadian profile of CGRP production in hypertensive rats has an inverse relationship with the profile of BP with its lowest plasma level at nighttime, and suggesting that CGRP together with the EETs could play a modulator role in vascular tone and be involved in circadian clocks of CHI and BRS. Relatively high plasma circadian level of CGRP and EETs associated with lower diurnal level of vasoconstrictor component of endothelial system in SO rats suggests a prevalence of circadian release of vasodilatory substances in this group of animals as compared to hypertensive rats.

## Discussion

Misalignment of circadian rhythms has been indicated as a causal factor for altered heart rate, blood pressure, and hormonal levels, and circadian changes in the autonomic nervous system activity play an important role in the control of cardiovascular function [1,2,3,4,5,7,9,14,19,29,43,44,45,46,47,48,49]. Investigations in spontaneously hypertensive rats provided evidence that the higher BP and heart rate during the dark period are likely related to the nocturnal behavior activity of the rats during which sympathetic activity and release of plasma pressor hormones is higher, which contributes to higher blood pressure. In contrast, during the light period, cardiovascular parameters are lower in animals at rest or asleep [22,23]. Our results are consistent with findings of the above mentioned authors. The shifted acrophases of hemodynamic parameters in 8-week hypertensive rats to late night hours in comparison to 4-week 1K-1C AH could be explained by a more significant increase in sympathetic tone and reduction in BRS, as a homeostatic buffer mechanism involved in major manifestations of sympathetic–parasympathetic interaction [45]. There are controversial findings related to CGRP’s role in AH [21,47,48,49]. In experimental studies it was demonstrated that CGRP acts through a compensatory depressor mechanism by partially attenuating a BP increase in drug (deoxycorticosterone acetate salt or N(G)-Nitro-L-arginine-methyl ester)-induced hypertension [24]. The vasodilatory mechanism of CGRP involves adenylyl cyclase stimulation with a resulting rise in cAMP, leading to relaxation. The studies suggest that CGRP plasma level undergoes circadian variability with its nocturnal plasma concentration rise that is involved in complex regulation of BP homeostasis [20,21,22,27]. The nocturnal rise in CGRP concentration was significantly lower in the hypertensive group in comparison to the control group and coincided with the blood pressure and heart rate fall [22]. In clinical studies plasma CGRP level wasn’t significantly increased in patients with secondary hypertension and was decreased after treatment suggesting the possible compensatory role of the production of CGPR in response to elevation in arterial pressure. Presumably, CGPR release is increased in the early stage of hypertension, having compensatory or protective significance [27]. Our results are in agreement with these findings, because the reduction in CGRP nocturnal release was more markedly expressed in 8-week 1K-1C model of hypertension, indicating more significant removal of CGRP through a compensatory depressor mechanism probably due to the regulatory role of CGRP circadian fluctuation in the development of arterial hypertension [22,27]. Disturbed endothelial function could be involved in the early hypertensive process that may be present before the alteration in basal sympathetic/parasympathetic tone, which was shown in clinical studies with increased basal concentration of plasma ET1 in borderline hypertension [8,11]. The involvement of ET1 in diurnal variation in blood pressure and arterial stiffness in patients with chronic kidney disease was observed by some authors [10]. In salt-sensitive hypertensive patients with low plasma renin activity, sodium depletion is associated with enhanced plasma responds of ET1 with a rise in plasma catecholamines level. These findings suggest a strong relationship between the sympathetic system, sodium sensitivity and endothelin production resulting in arterial pressure increase in these patients. In our experiments, circadian ET1 plasma fluctuation was revealed in both groups of salt-sensitive hypertension. Peak values were more significantly expressed in 8-week hypertensive rats that corresponded to acrophases of BP and circadian reduction at 16:00 h in contrast to acrophases established for CCGP. These findings are in accordance with the results that showed increased basal level of ET1 and decreased level of CGRP in the hypertensive state [20,49], suggesting the diurnal variability of ET1 and CGRP circadian production and their involvement in diurnal fluctuation of CHI. EETs, as endothelium–derived hyperpolarizing factor, stimulate large-conductance Ca^2+^ – activated potassium channels (KCa^++^) mediating nitric oxide and prostaglandin independent vasodilation that opposes the vasoconstrictor actions of angiotensin II (Ang II) and through epoxygynase pathways provide significant influence in cardiovascular disease [25,26,27,46]. The circadian profile of EETs release in hypertensive animals demonstrated the lowest plasma level at 24:00 h, while ET1 peak level at this night period approximated the acrophases of BP that indicated the lack of circadian nocturnal production of EETs in both groups of animals with more marked reduction in their plasma level in 8-week 1k-1C AH. Other studies have demonstrated that EETs can oppose vasoconstrictor stimuli to maintain proper vascular tone [48].

Circadian acrophases for EETs and CGRP plasma concentrations were revealed at the same period of time 16:00 h in the two groups of hypertensive rats, with diurnal reduction of their plasma concentration at 24:00 h that coincide with corresponding circadian changes in CHI and BRS in contrast to ET1. Our results are in agreement with experimental data showing significant attenuation of K^+^ stimulated and capsaicin-evoked CGRP release from trigeminal ganglion neurons after pretreatment with 14,15–EETs inhibitor EEZE, suggesting that EETs could act as intracellular regulators of neuropeptide production and close relationship between EETs and CGRP circadian release [49] which is important for treatment of salt-sensitive resistant arterial hypertension. For avoiding complications associated with elevated BP and improving endothelial function, levels of CGRP, EETs and especially ET1 may be used as a potential biomarker for the restoration of a normal circadian BP pattern in salt-sensitive forms of hypertension and in prehypertensive state in persons undergoing chronic stress stimuli predisposing to the development of arterial hypertension.

## Conclusion

Ours results suggested that the development of vasorenal 1K-1C model of hypertension may cause circadian alterations in CHI and BRS involving diurnal variability in ET1, CGRP and EETs production and support the concept that stable CGRP agonist, EETs analog and especially ET1 antagonist could have preventive and therapeutic potential. The obtained results give ground to personalized antihypertensive chronotherapy in different salt-sensitive forms of hypertension.
